# Impact of the COVID-19 Pandemic on Latino Families With Alzheimer Disease and Related Dementias: Qualitative Interviews With Family Caregivers and Primary Care Providers

**DOI:** 10.2196/42211

**Published:** 2024-03-08

**Authors:** Jaime Perales-Puchalt, Jill Peltzer, Monica Fracachan-Cabrera, G Adriana Perez, Mariana Ramírez, K Allen Greiner, Jeffrey Murray Burns

**Affiliations:** 1 Department of Neurology, University of Kansas Alzheimer’s Disease Research Center, Fairway, KS, United States; 2 Department of Family Medicine, University of Kansas Medical Center, Kansas City, KS, United States; 3 School of Nursing, University of Pennsylvania, Philadelphia, PA, United States

**Keywords:** Latino, dementia, caregiving, COVID-19, Alzheimer’s disease, health disparity, qualitative research, transcript analysis, health inequality, minority population, epidemiology, peer support, social services, health care, Alzheimer’s, minority, qualitative, interview, caregiver, primary care, impact, resilience, disparity, outcome, Alzheimer disease, Alzheimer

## Abstract

**Background:**

Latino individuals experience disparities in the care of Alzheimer disease and related dementias (ADRD) and have disproportionately high COVID-19 infection and death outcomes.

**Objective:**

We aimed to gain an in-depth understanding of the impact of the COVID-19 pandemic among Latino families with ADRD in the United States.

**Methods:**

This was a qualitative study of 21 informal caregivers of Latino individuals with ADRD and 23 primary care providers who serve Latino patients. We recruited participants nationwide using convenience and snowball sampling methods and conducted remote interviews in English and Spanish. We organized the transcripts for qualitative review to identify codes and themes, using a pragmatic approach, a qualitative description methodology, and thematic analysis methods.

**Results:**

Qualitative analysis of transcripts revealed eight themes, including (1) the pandemic influenced mental and emotional health; (2) the pandemic impacted physical domains of health; (3) caregivers and care recipients lost access to engaging activities during the confinement; (4) the pandemic impacted Latino caregivers’ working situation; (5) the pandemic impacted health care and community care systems; (6) health care and community care systems took measures to reduce the impact of the pandemic; (7) Latino families experienced barriers to remote communication during the pandemic; and (8) caregiver social support was critical for reducing social isolation and its sequalae.

**Conclusions:**

Latino families with ADRD experienced similar but also unique impacts compared to those reported in the general population. Unique impacts may result from Latino individuals’ underserved status in the United States, commonly held cultural values, and their intersectionality with ADRD-related disability. Family caregiver social support was crucial during this time of adversity. These findings suggest the need for more equitable access, culturally appropriate and trustworthy content and delivery of health care and community services, as well as stronger financial and social supports for family caregivers.

## Introduction

Alzheimer disease and related dementias (ADRD) have a devastating impact on Latino families. Compared to non-Latino White individuals, Latino individuals experience disparities in ADRD risk, detection, treatment, and care. While Latino individuals are 1.5 times more likely to have ADRD [1], they are 1.4 times less likely to be diagnosed [2]. Latino individuals are diagnosed with an 8-month delay [3], are underprescribed cholinesterase inhibitors [4], and underuse ADRD support services [5]. These disparities are further compounded by Latino individuals’ lower ADRD knowledge and inclusion in ADRD research [6-8].

Latino families have also been disproportionately impacted by the COVID-19 pandemic. As of January of 2022, among individuals aged 65 years and older, Latino individuals represent 8% of the US population but 13% of COVID-19 cases [9]. Latino individuals of any age are also more likely to be infected with COVID-19, representing 18% of the US population but 25% of those infected. When controlling for age, the percentage of COVID-19 deaths is also higher among Latino individuals. For example, Latino individuals represent 14% of the deaths among those aged 65 years and older, which is nearly twice as much as their representation in the US population aged 65 years and older [9]. Latino individuals are also less likely to be vaccinated against COVID-19, representing only 12% of those initiating vaccination [10]. Some factors contributing to these disparities include having a job considered essential, living in a segregated geographic area, living in overcrowded households, limited English proficiency, and reduced use of preventive behaviors to avoid COVID-19 infection [11].

Despite the growing literature on the impact of COVID-19 among Latino individuals, little is known about the impact of COVID-19 on Latino families with ADRD. Studying COVID-19’s impact on Latino families with ADRD is important given that Latino individuals are populations with disparities in both ADRD and COVID-19, both of which might synergistically impact their health [9,10,12]. A study in California that included 8 Latino individuals with cognitive impairment found that the biggest impact of COVID-19 on these and other underserved communities included the fear generated by the pandemic, distress stemming from feeling extremely isolated, and receiving inaccurate information about COVID-19 from different sources [13]. The study also reported some strategies participants used for coping during the pandemic (eg, mask wearing and remote communication) and the importance of access to essential resources such as friends, the church, and local programs. Although this work provided important insights on the impact and resilience factors for diverse populations with cognitive impairment, it is crucial to increase the Latino representation for a more detailed understanding. It is also important to listen to the perspectives of family caregivers and primary care providers (PCPs), who may provide a different point of view and allow triangulation. Given the central role of family in Latino culture [14] and PCPs in the health care system [15], the perspectives of family members and PCPs can provide privileged insight into the experiences of persons with ADRD, irrespective of their level of cognitive impairment, their family, and their interaction with the health care system.

The aim of this study was to gain an in-depth understanding of the impact of the COVID-19 pandemic among Latino families with ADRD. To achieve this goal, we interviewed family caregivers and PCPs who serve Latino individuals with ADRD across the United States. Findings can inform actions to help Latino families with ADRD remain safe while maintaining a good quality of life during this ongoing pandemic as well as future ones.

## Methods

### Assessment

This study used a qualitative descriptive design. Qualitative studies rely on text data and aim to understand the meaning of human action [16]. The current COVID-19–focused analysis is part of a broader study that planned to identify what ADRD care services are offered in primary care and how they are delivered across a variety of settings. This study was informed by the *National Institute on Minority Health and Health Disparities Research Framework*, which considers the complex and multifaceted nature of minority health and health disparities [17]. This framework includes different domains of influence (biological, behavioral, physical or built environment, sociocultural environment, and health care system), as well as different levels of influence (individual, interpersonal, community, and societal) within these domains.

### Ethical Considerations

The data collected were stripped of all identifying information and labeled with a code. The code can be linked to the individual’s identity, but the key linking the code to the identifying information is held separately from the research data. The University of Kansas Medical Center Institutional Review Board approved this project (STUDY00145615). Before the interview, all participants completed an informed written consent on the web either via their computers, tablets, or phones. The research team compensated participant’s time by posting them a US $40 gift card.

### Sample

We recruited 2 groups of participants: (1) family caregivers of people living with ADRD and (2) PCPs. Inclusion criteria for caregivers included being aged 18 years or older, identifying as a close friend or relative of a Latino person with ADRD diagnosed by a health care provider or research study, providing or having recently provided care to them at least once a week in person or via phone, being proficient in English or Spanish, living in the United States, and being willing to participate in the study. Inclusion criteria for PCPs included being a medical doctor, doctor in osteopathic medicine, nurse practitioner, or physician assistant who currently or recently provided primary care services to Latino families with ADRD in the United States.

Caregivers were recruited via convenience sampling from diverse sources, including a research registry, clinics, and community ADRD patient lists, internet and newspaper advertisements, and connections with community partners and research team members. PCPs were initially recruited using snowball sampling techniques: first by contacting connections of the research team and later asking interviewed PCPs for referral of other PCPs.

We used purely qualitative semistructured interviews, which allow for the comparison between participants while allowing spontaneous exploration of topics relevant to unique participants [18]. The interviews took place between November 2020 and April 2021. All but 2 interviews were conducted via secure videoconference. The other 2 interviews were conducted over the phone. The interviewer used 2 audio recorders to reduce the risk of data collection failure and took notes at the end of each interview to summarize the findings. Questions were not provided to participants before the interview. No one else besides the interviewer and the participant was present during interviews, although in a few instances, participants had to pause the interview briefly to address issues with their loved ones (caregivers) or colleagues (PCPs).

The process for each interview was similar: JP-P interviewed all participants. The interview started with a short conversation aimed at developing rapport and explaining the main goals of the interview. The first questions asked about the basic characteristics of the participants. Core questions of the survey asked about participants’ experience with primary care clinics (caregivers) and serving Latino individuals with ADRD (PCPs). Unless participants had mentioned it spontaneously, the interviewer asked participants halfway through the interview how the COVID-19 pandemic had impacted them, their care recipient (caregivers), and the Latino families with ADRD they serve (PCPs; see Multimedia Appendix 1 for the interview guides). The interview questions had been previously pilot-tested within the team. The interviewer audiotaped all interviews, which were designed to last 45-60 minutes, including the COVID-19 questions. Interviews were conducted in English or Spanish, and those with caregivers included simple language to account for different levels of literacy. A professional team transcribed all interviews and the interviewer reviewed them for accuracy.

To bring rigor and validity to the research process, the interviewer used active listening techniques during the interview aimed at confirming the information shared by participants. The potentially lower theoretical ADRD expertise by family members and PCPs could create a perceived power differential with the interviewer. In fact, some family members and PCPs expressed worry about the interviewer potentially testing their ADRD knowledge. To reduce this perception of power differential, the interviewer emphasized that the interviews would ask about their experiences and that they were the experts in those experiences. JP-P already knew some of the family caregivers via his research but knew none of the PCPs. To balance how much participants knew about him, he introduced himself, his background, previous work, and research goals the first time he contacted them and at the beginning of the interview. Both coders (JP-P and MF-C) had previous coding experience. The fact that only 1 coder conducted the interviews and that they both had different backgrounds (JP-P: PhD, man, faculty, and psychology and public health education vs MF-C: MD, woman, research associate, and medical education) allowed different perspectives.

### Data Analysis

We organized the transcripts and notes for qualitative review, using a pragmatic approach and thematic analysis methods, which emphasize identifying, analyzing, and interpreting patterns of meaning within qualitative data [19,20]. We organized the data in Dedoose [21]. JP-P identified relevant sociodemographic, ADRD relationship, and clinical service data in the interviews and summarized it into descriptive statistics. JP-P and MF-C independently read the interviews and notes once initially to familiarize themselves with the data, coded the content of the text by identifying codes and themes, and resolved coding disagreements through discussion and consensus.

## Results

### Characteristics of the Sample

Considering the time-sensitive nature of this work, we decided to stop recruitment when data saturation was achieved at 44 participants. Table 1 shows the characteristics of the 21 family caregivers and 23 PCPs. Most caregivers were women (19/21, 90%), younger than 66 years (16/21, 76%), and children of the care recipient (15/21, 71%), most of whom had Alzheimer disease (14/21, 67%). All but 1 (20/21, 95%) participant identified as Latino of diverse origins; 6 (29%) were born in the United States; and all lived in urban regions in the Midwest (15/21, 71%), the Northeast (5/21, 24%), and the South (1/21, 5%). Interviews were conducted in Spanish with 13 (62%) caregivers, 14 (67%) of them reported good English proficiency, and 8 (38%) reported that their care recipient had good English proficiency.

**Table 1.** Characteristics of the sample.

Characteristics		Family caregivers (n=21)	Primary care providers (n=23)
Women, n (%)		19 (90)	13 (57)
**Age group (years), n (%)**			
	33-65	16 (76)	N/A^a^
	66+	5 (24)	N/A
**Ethnoracial group, n (%)**			
	Latino	20 (95)	10 (43)
	Non-Latino Asian	0 (0)	1 (4)
	Non-Latino Black	0 (0)	2 (9)
	Non-Latino White	1 (5)	10 (43)
**Subgroup among Latino participants, n (%)^b^**			
	Caribbean	3 (14)	3 (13)
	Central American	4 (19)	1 (4)
	Mexican	10 (48)	2 (9)
	South American	3 (14)	3 (13)
US born, n (%)		6 (29)	12 (52)
Live in an urban setting, n (%)		21 (100)	18 (78)
Interviewed in Spanish, n (%)		13 (62)	5 (22)
**Region, n (%)**			
	Midwest	15 (71)	15 (65)
	Northeast	5 (24)	2 (9)
	West	0 (0)	4 (17)
	South	1 (5)	0 (0)
	Puerto Rico	0 (5)	2 (9)
Good English proficiency, n (%)		14 (67)	N/A
Can provide services in Spanish, n (%)		N/A	12 (52)
**Relation to care recipient, n (%)**			
	Child	15 (71)	N/A
	Spouse	5 (24)	N/A
	Friend	1 (5)	N/A
**Diagnosis of care recipient, n (%)**			
	Alzheimer disease	14 (67)	N/A
	ADRD^c^ (unspecified)	2 (10)	N/A
	Mild cognitive impairment	2 (10)	N/A
	Early onset Alzheimer disease	1 (5)	N/A
	Early onset Alzheimer disease and frontotemporal dementia	1 (5)	N/A
	Parkinson dementia	1 (5)	N/A
More than 1 care recipient, n (%)		1 (5)	N/A
Care recipient has good English proficiency, n (%)		8 (38)	N/A
**Type of provider, n (%)**			
	Medical doctor	N/A	15 (65)
	Nurse practitioner	N/A	7 (30)
	Physician assistant	N/A	1 (4)
**Type of clinic, n (%)**			
	Private	N/A	11 (48)
	Safety net and federally qualified	N/A	8 (35)
	Academic	N/A	4 (17)
**Percent of Latino patients, n (%)**			
	Less than 29%	N/A	7 (30)
	30%-74%	N/A	7 (30)
	75%+	N/A	9 (39)
Total minutes of recording, range		25-61	35-73

a N/A: not applicable.

b Only 9 Latino primary care providers shared subgroup information.

c ADRD: Alzheimer disease and related dementias.

More than half of PCP participants were women (13/23, 57%) and US born (12/23, 52%). A total of 10 (43%) PCPs identified as Latino of diverse origins and another 10 (43%) identified as non-Latino White. A total of 18 (78%) PCPs lived in urban settings, with the Midwest being the most common region (15/23, 65%). A total of 12 (52%) PCPs reported being able to communicate with their patients in Spanish. In all, 15 (65%) PCPs were medical doctors and 7 (30%) were nurse practitioners; the types of clinics they practiced in varied, along with a wide distribution of the percentage of Latino patients served.

### Themes

We developed 8 themes from the analysis. Table 2 shows the themes and codes identified in the qualitative analyses.

**Table 2.** Themes and related codes.

Theme	Code
1. The pandemic influenced mental and emotional health	1.1. Fear of infection 1.2. Uncertainty 1.3. Distress 1.4. Depression
2. The pandemic impacted physical domains of health	2.1. Accelerated deterioration 2.2. Poor nutrition 2.3. Death
3. Caregivers and care recipients lost access to engaging activities during the confinement	3.1. Isolation 3.2. Deprivation
4. The pandemic impacted Latino caregivers’ working situation	4.1. Avoiding exposure to infection 4.2. Disrupted work 4.3. Increased work burden
5. The pandemic impacted health care and community care systems	5.1. Health care systems 5.2. Community care systems
6. Health care and community care systems took measures to reduce the impact of the pandemic	6.1. Home health 6.2. Remote communication
7. Latino families experienced barriers to remote communication during the pandemic	7.1. Low trust 7.2. Insufficient skills and diminished abilities 7.3. Inadequate support
8. Caregiver social support was critical for reducing social isolation and its sequalae	8.1. Protecting from COVID-19 infection 8.2. Reducing psychosocial impact 8.3. Reducing health care and community care system impact

#### Theme 1. The Pandemic Influenced Mental and Emotional Health

The mental and emotional health domains described by caregivers and PCPs included fear of infection, uncertainty, distress, and depression. This was the most reported impact by all interviewees.

##### 1.1. Fear of Infection

The fear of COVID-19 infection for the care recipient was reported by most of the caregivers and PCPs. While some caregivers mentioned that they or their care recipients had not been infected, they were fearful about the risk of COVID-19 to the care recipient, who was at risk for significant disease sequalae. Other caregivers or their care recipient were experiencing COVID-19 at the time of the interview. PCPs explained that this fear was especially felt by minoritized individuals including Latino individuals, given the news that they were getting infected and dying at disproportionate rates compared to non-Latino White individuals. These 2 example quotations summarize this fear:

Most ADRD patients are old and this is the population at the highest risk, and they are even more scared.

They do not want to come in. The news said very early that ethnic minorities were getting more severe disease that they just stopped coming in and it is really hard to get a hold of them.

##### 1.2. Uncertainty

According to both caregivers and PCPs, caregivers and care recipients had feelings of uncertainty. There was uncertainty about when they would be eligible to get the COVID-19 vaccine, when the caregiver would get some respite by being able to take the care recipient back to the senior center, confusion about what was happening due to cognitive impairment, and general uncertainty about their lives. One caregiver defined their lives during the pandemic succinctly as follows:

Everything has been in limbo with COVID.

##### 1.3. Distress

Heightened fear and daily uncertainty led to increased levels of recurring distress. On top of pandemic-related distress, caregivers were unable to access caregiver support services and the respite they needed to manage their own health. They also lacked access to the care residences needed to provide the safest care for the care recipients. This distress added on top of the other stresses Latino families often experience, as this caregiver explained:

I was recently told I had cancer. Now I need to get some further testing. This affected me a lot too. It’s also been hard to work, take care of the house, my kids…because when it’s not one problem it’s another. It’s been really hard…so much stress.

##### 1.4. Depression

Caregivers and PCPs highlighted the frequency and severity of depressed mood among caregivers and care recipients, especially during the lockdown due to the lack of social support and increased social isolation. Two PCPs noted the following:

All of my ADRD patients are sad…they are isolated, by themselves.

The social isolation of COVID has caused a significant level of depression.

A caregiver also shared the following:

I’m very sad, depressed, neurotic…I’ve no patience…very little patience. To me, life has changed a huge deal…360 degrees.

### Theme 2. The Pandemic Impacted Physical Domains of Health

The physical domains described by caregivers and PCPs included accelerated deterioration, poor nutrition, and death.

#### 2.1. Accelerated Deterioration

Caregivers and PCPs noticed accelerated cognitive and physical deterioration. One of the caregivers had to move their loved one to a long-term care facility in the midst of the pandemic and saw a rapid decline in cognition. Another PCP reported how the lockdown resulted in reduced access to treatment among some patients, leading to accelerated cognitive and physical decline. The caregivers and PCPs attributed decline to social isolation and a lack of engagement in activities that affected mood, such as depression, sadness, and apathy. A caregiver explained her mother’s memory decline during lockdown as follows:

I was thinking her memory and abilities declining all the time she was locked down here, because of the cold weather and COVID might have affected her more.

#### 2.2. Poor Nutrition

Food access was a critical issue, leading to poor nutrition that contributed to accelerated declines in health. Reasons for poor food access included delays in the mailing system and financial insecurity. These barriers interacted with care recipients’ impaired abilities and male caregivers’ lack of cooking skills and resulted in a frequent use of unhealthier options due to their accessibility. For example, a male caregiver explained that their access to healthy food was impacted because his spouse could not cook anymore due to ADRD:

…and my cooking abilities are not all that great…so fast food has been our life for the last year maybe even more because she has not been able to cook for some time.

#### 2.3. Death

Participants reported death due to COVID-19 among their Latino families. For example, a PCP explained that several of his Latino patients died during their yearly visit to their home country—deaths that would not have occurred prior to the pandemic. A caregiver explained how a relative with ADRD died shortly after being moved to a residential facility:

He did not live too long after that, he literally stopped eating…so eventually he was put on Morphine because he was in a lot of pain from multiple things and that is how he passed.

### Theme 3. Caregivers and Care Recipients Lost Access to Engaging Activities During the Confinement

Even though participants understood the need for the lockdown, they frequently described how it contributed to social isolation and deprivation, which, in turn, reduced engagement in activities and led to depression and apathy.

#### 3.1. Isolation

The pandemic paralyzed or slowed down operations in clinics, church services, caregiver support activities, senior centers, and residences. Additionally, PCPs, home health assistants, residence staff, and family members were infected. In fact, a caregiver’s former PCP who spoke Spanish had died of COVID-19 and was replaced by a non-Spanish speaker. One of the PCPs mentioned the following:

I think it is really a negative impact and really socially isolating a lot of people, which as a PCP always makes me worried because of how much stress that adds to people.

#### 3.2. Deprivation

Caregivers also reported that their care recipient and themselves lost access to engaging activities during the confinement, such as visiting with their peers, going out to eat, and attending social events at the local senior center. A caregiver summarized this feeling as follows:

We have been so stuck with this life of COVID not being able to do anything or go anywhere.

### Theme 4. The Pandemic Impacted Latino Caregivers’ Working Situation

The pandemic’s impact on Latino caregivers’ working situation included avoiding exposure to infection, disrupted work, and increased work burden.

#### 4.1. Avoiding Exposure to Infection

Families had to make difficult choices between exposing frail, older members to COVID-19 or losing critical income to support the family. A PCP explained how it affected Latino families negatively:

I have a large patient population that works in the meat packing plant industry, and they were really scared of COVID infection due to an outbreak they experienced.

#### 4.2. Disrupted Work

While this interruption gave some the opportunity to care for their loved ones, many caregivers’ inability to work had a significant impact on the family’s financial situation. This impact also generated family conflicts, such as the following from a caregiver who quit her job abroad to move back with her care recipient:

I ended up fighting with my sister, who, like me, was also living abroad because I ended up moving with Mom. I told her, who will care for Mom…our Mom?

#### 4.3. Increased Work Burden

Some Latino caregivers were considered essential workers during the COVID-19 pandemic, which increased their work burden and risk of infection. A PCP mentioned the following with respect to their Latino patients, including relatives of people with ADRD:

Many of them work in restaurants and similar jobs that have been affected by it. In construction, people have had no issues, but they have a lot more work.

### Theme 5. The Pandemic Impacted Health Care and Community Care Systems

The pandemic impacted multiple systems that support care recipients and caregivers, including health care and community care systems.

#### 5.1. Health Care Systems

The pandemic impacted the availability and quality of services provided to Latino families with ADRD in primary, neurology, and long-term care. Services included educating Latino caregivers on ADRD, assessing for dehydration, creating rapport, and the treatment and assessment of ADRD and other conditions. PCPs had to reduce physical contact with care recipients, which reduced their chance to convey warmth to their patients. An example of how the pandemic affected services includes a PCP who said the following despite being able to see patients again after the lockdown:

It’s not the same, you no longer have the closeness or attachment with the patient, I can no longer touch their shoulders or give them a hand because now we have to keep our distance.

A caregiver complained about the poor prioritization of his primary care clinic with respect to obtaining the COVID-19 vaccine for people with ADRD:

I got an e-mail…from the hospital saying that if I wanted the COVID shot to call and make an appointment, and…I said what about my wife…she needs it more than I do.

The novelty of COVID-19 also increased confusion between COVID-19 and ADRD symptoms in primary care and neurology clinics. A caregiver mentioned the following:

Both the PCP and the neurologist saw the issue as part of the ADRD, not as part of the virus…But I’m sure it was the virus because she could swallow again after two or three weeks, and she was doing better than before.

PCPs mentioned their patients with ADRD acquiring COVID-19. As an extreme case, 1 PCP who serves institutionalized patients, which include several Latino patients, reported the following:

One of my nursing homes right now, I think we are up to 22 positives today out of 40 patients.

#### 5.2. Community Care Systems

The access and quality of services provided by community care systems to Latino families with ADRD were also impacted by the pandemic. These included services provided by senior centers, home assistants, caregiver support groups, and home-delivered meals. Two caregivers described the following:

The senior center opened two weeks ago. But I told them I’d have her at home. There’s no need to expose her to anything…Four hours are not going to make a difference and it’s ultimately going to give me more work. And she would be exposed. I don’t trust that the protocols are ideal for her to go.

The reason why I’m not going to support groups now is initially because of COVID. They had stopped and I had not heard that they are doing anything with the group again.

Speaking about their Spanish-speaking home assistants, a caregiver reported the following:

With COVID, this has been terrible, because people don’t want to work. Mom had two that got COVID.

Some Latino families with ADRD we interviewed tried to use home-delivered meals for the first time during the pandemic to reduce risk of infection. However, home-delivered meals services were not culturally prepared to serve Latino families, as this caregiver explained:

We had Meals on Wheels, but we just could not have those meals, they were terrible. They were terrible for a Latino family.

### Theme 6. Health Care and Community Care Systems Took Measures to Reduce the Impact of the Pandemic

Families and PCPs highlighted the impact Latino families with ADRD experienced and the reduced access and quality of health care and community care services. In response, these services adapted to the COVID-19 pandemic context by leveraging home health and remote communication.

#### 6.1. Home Health

Most senior centers were closed during the pandemic, but some reopened shortly after the lockdown was lifted. Clinics and other institutions leveraged home care professionals to provide care for their patients. These included private sources but also all-inclusive care programs for older adults and Catholic charities. Regarding the all-inclusive care programs for older adults, a caregiver and a PCP mentioned the following, respectively:

The home care services come from an all-inclusive care program for the elderly. I think since August from last year they’ve been coming every day from 8 AM to 12 PM, which is when I have more work online meetings.

Now if they need to be seen we send people to the home and we can do that. If somebody needs to be seen we will send a provider, a nurse, whoever else needs to see them.

#### 6.2. Remote Communication

Participants and PCPs stressed their increased use of remote communication during the pandemic. Purposes of remote communication included conducting telehealth visits among clinicians, caregivers, and care recipients; using interpreters; and ordering medications. The communications methods used included phone and video calls, as well as emails and patient portals. A PCP exemplified this shift as follows:

That’s the way I’ve communicated with patients during the pandemic…over the phone or virtually and ordering their medications directly electronically to the pharmacy or the lab.

### Theme 7. Latino Families Experienced Barriers to Remote Communication During the Pandemic

As mentioned earlier, remote communication became paramount during the COVID-19 pandemic for health care and community care services. Remote communication also became an important tool for families. However, Latino families with ADRD experienced barriers in communication related to their low trust, insufficient skills, diminished abilities, and inadequate support.

#### 7.1. Low Trust

Participants identified trust of the remote communication source as a barrier, specifically among the Latino families they serve. A PCP said the following:

I have seen specifically in the Latino population they are not quite as engaged with some of the technology and telehealth, I think they are a little bit worried about how to use it or ‘is my information safe?’.

#### 7.2. Insufficient Skills and Diminished Abilities

Another barrier to remote communication was the low technology savviness of Latino individuals and the diminished abilities of care recipients due to their ADRD. These included cognitive or physical impairment. The relative of a care recipient said the following:

It is really hard to Facetime with him unless there is somebody right there with him because the camera keeps going to the ceiling or he does not want to talk to me, so having a Facetime with him is sometimes difficult.

#### 7.3. Inadequate Support

Families highlighted the need for support to communicate remotely, which was hindered by technological difficulties (eg, patient portals not being in Spanish and translators being harder to understand over the phone vs in person), low access to devices, and a lack of family involvement. A family caregiver who limited her visits to reduce the risk of infecting her care recipient mentioned the following:

I try to keep the peace with my brother, so I gave Mom an iPad, but she does not know how to use it and she needs help using it and my brother who lives with her is reluctant to facilitate the Facetime calls.

### Theme 8. Caregiver Social Support Was Critical for Reducing Social Isolation and Its Sequalae

Caregivers were crucial in protecting care recipients’ health and maintaining their quality of life during the pandemic. In fact, caregiver support was the most frequently mentioned type of social support. Caregivers largely supported care recipients in protecting against COVID-19 infection and reducing the psychosocial, health care, and community care impact of the COVID-19 pandemic.

#### 8.1. Protecting Against COVID-19 Infection

Caregivers made sure that they and their care recipients would wear face masks and shields, wash their hands often, keep a safe distance from others (or themselves if the caregiver was exposed to others), and get tested for COVID-19. Caregivers took over some tasks they typically did not do, to increase the care recipient’s safety and physical health, including taking care of medicines, monitoring nutrition, planning activities, and going shopping alone. Caregivers were proactive in acquiring vaccinations as soon as they were available for themselves and their loved ones, despite systemic barriers, which allowed them to provide care for their care recipient rather than avoiding contact. Two caregivers described how they advocated for their loved ones to get vaccinated as soon as possible:

I feel like if I had not been persistent and kept calling, she probably would still not have her vaccines.

I do not understand how such an old person has not gotten a call from her primary care clinic…So, I asked a nun at a catholic hospital at a different state if she could give permission for my wife to go, and she did, so my wife got vaccinated in that other state.

#### 8.2. Reducing Psychosocial Impact

Social support was critical for reducing loneliness and its consequences. Caregivers sought informal sources of support, including family and friends, and formal sources, such as clinical resources and respite care. Many adult child caregivers either moved in with the care recipient or moved the care recipient to live with them to provide daily care. There were only few cases where a caregiver reported not providing family support to the care recipient. Two caregivers’ quotes exemplified the importance of their social support to reduce the care recipients’ loneliness and the mental health impact of the COVID-19 pandemic:

I am the only one that visited her and would take her anywhere. No one has visited ever since I moved back. My sister might have visited once, maybe.

When I am not involved in calling my mother often, she is just very anxious, so I think calling multiple times a day everyday helps.

#### 8.3. Reducing Health Care and Community Care System Impact

Besides advocating to get their loved ones vaccinated, family caregivers had to solve additional problems related to the health care and community care systems. These problems included assisting with remote communications with their providers, requesting that their loved one’s cognition is assessed, or requesting that they be allowed to accompany the care recipient into health care visits at a time when these were forbidden due to the pandemic. Two caregivers said the following:

The PCP told me those were side effects of COVID and that little by little her memory would come back…Every three months, I’d go to her and insist, until a neurologist saw her and diagnosed her with ADRD.

They wouldn’t let me come in with Mom, but I explained that she would not be able to understand anything due to her memory, and after some discussion, they let me in too…I convinced them despite my poor English proficiency.

## Discussion

### Principal Findings

This study aimed to gain an in-depth understanding of the impact of the COVID-19 pandemic among Latino families with ADRD, from the perspectives of family caregivers and PCPs. To achieve this goal, we interviewed 21 family caregivers of Latino individuals with ADRD and 23 PCPs across the United States. These participants were diverse with respect to their region, primary language, and other characteristics. We found that the COVID-19 pandemic has impacted Latino families with ADRD at multiple levels, ranging from physical to health care and community care levels. Latino family caregivers’ actions were key to addressing these new challenges.

### Comparison With Prior Work

Some impacts of the COVID-19 pandemic on Latino families with ADRD resembled impacts reported in previous research for the general population. For example, accelerated cognitive deterioration was also reported in a mixed methods study in the United Kingdom where 184 caregivers and 24 people with ADRD were interviewed [22]. The lack of access to engaging activities and human contact were also reported in the UK study, which were found to be potential drivers of the accelerated deterioration. Other similar findings from this study include the impact on care recipients’ and caregivers’ mental health, the barriers with the technology needed to replace in-person contacts, and the importance of caregivers to bring about resilience despite the adversities. Similar impacts have also been reported among family caregivers of people with ADRD in India and Italy [23,24]. Despite these similarities, our findings highlight the need to pay attention to pandemic-related stress that add up with many other life stresses found frequently in minoritized populations [25], as well as additional factors that compound the already existing digital divide’s impact on remote communication among Latino individuals: low trust in these technologies and decline in technological abilities among people with ADRD [13,23,24].

Some impacts have been reported less frequently or not reported at all previously. To our knowledge, no study has reported the impact of the COVID-19 pandemic on nutrition among people with ADRD and their families. This impact is in line with a systematic review of 28 studies across the world, where most of them identified changes in food intake toward the adoption of unhealthy eating among people of all ages [26]. Our findings shed light on potential solutions for the Latino community, as those impacted tended to be male caregivers with low income and families for which the meal delivery services did not provide culturally appropriate meals.

We also found that the COVID-19 pandemic impacted caregivers’ work. This finding aligns with a study from India where many caregivers had to start working from home or faced disruptions that affected their finances [24]. In our Latino sample, the pandemic did not just disrupt work for some but also increased the work burden of others. Latino individuals may have been especially impacted by the work consequences of the pandemic, as they are overrepresented in the frontline workforce, tend to live in multigenerational homes, and have among the lowest average incomes in the United States [27-29].

Our findings align with 2 commonly reported factors among Latino individuals that have been shown to impact health behaviors, including health care use and assertiveness [30-32]. First, our interviews revealed that the news about Latino individuals’ disproportionate risk of COVID-19 infection and death [9] may have increased the fear among Latino families and contributed to their low use of sometimes critically needed resources. These findings align with “fatalism,” a belief that events are beyond one’s control [31]. Second, we also found that to reduce the risk of infection, PCPs reduced physical contact with their patients, which they saw as a barrier to care. This finding suggests an impact on “personalism,” or the importance of establishing relationships through warm interactions [31].

### Limitations

This study has some limitations. Remote recruitment and interviews increased the representation of participants in rural areas and other states. However, video calls and phone calls led to some communication issues, which, in some cases, reduced the amount of information we could collect and affected the quality of the audio. The inability to conduct in-person recruitment and interviews may have excluded the most underserved individuals, who could have been contacted via health fairs before the pandemic started. We did not interview individuals with ADRD, which did not allow a full triangulation between them, their caregivers, and PCPs. Although Latino caregivers tend to be women [33], they were overrepresented in our study, likely also due to women’s higher likelihood to participate in health-related research [34,35]. The sample size was relatively small and not probabilistic, which reduces the generalizability of the findings. We did not return the transcripts to participants for comment or correction. As with most studies, individuals who participated in the study were motivated to participate. We do not know how much their discourse compares to those who decided not to participate.

### Implications and Future Directions

This study has implications for public health. Given the efficacy of existing COVID-19 vaccines [36], ensuring access to ongoing boosters among Latino individuals with ADRD and their families will be needed. To do so, it will continue to be necessary to hold events at flexible times and days; provide care at convenient venues; and improve the communication with Latino individuals by using a wide range of communication modalities (eg, calling, texting, and patient portals) in a way that is trustworthy, easy to use for people with cognitive impairment, and linguistically and culturally appropriate. The common stress and depression related to the fear of COVID-19 infection, uncertainty, and confinement among Latino families with ADRD highlight the need improve access to mental health services in general and specifically during pandemics. An example of a potentially inexpensive intervention is layperson-delivered, empathy-oriented telephone call programs, which have been shown to reduce loneliness, depression, and anxiety [37]. These services can be provided by governments or charity organizations and offered via primary care clinics, health departments, and entities that identify those who are potentially in need. Other useful services may include cognitive and physical engagement activities to reduce confinement-related deterioration. Since stresses are cumulative and Latino individuals tend to have higher levels of socioeconomically driven chronic stress [25], building stronger and more accessible social and health care welfare systems will also be key.

The impact of the pandemic on physical and cognitive decline, potential sources of support, and the crucial importance of families in the care of Latino individuals with ADRD highlight the need to provide them with financial support for their services, respite when possible, and accessible caregiving support training. Home care services have been essential in caring for some Latino individuals with ADRD and providing respite to their caregivers. However, these professions tend to be poorly paid, and their services are hard to access. Home care workers’ salary could be adjusted to the value of their service and covered by health insurance companies or government programs.

Our findings can inform future studies. Our findings regarding the physical and cognitive deterioration caused by the pandemic and the importance of family support may help inform future studies on the differential impact on ethnic groups in community and institutional settings and on the buffering effect of social support and health care and community care services. For example, nursing homes that have a higher proportion of ethnically minoritized populations had higher rates of COVID-19 infection and death [38]. Studies could explore whether this disproportionate impact also applies to cognitive and functional decline. Our data suggest that Latino families where men were the primary caregiver were at risk of poor nutrition and that home meal delivery services not offering culturally appropriate meals was a barrier to improving nutrition quality. Future studies can develop remote interventions to provide education on how to cook healthy, culturally appropriate meals for Latino family caregivers and explore the cost-effectiveness of offering Latin American healthy meals in communities where Latino individuals are present. Our findings also suggested that the awareness that Latino individuals had a higher infection and death risk from COVID-19 might have reduced their use of health care services. Future studies should develop culturally appropriate messaging in response to disparities where the fear and related factors facilitate health behaviors instead of reducing them.

### Conclusion

In this study, we have found that the COVID-19 pandemic has impacted Latino families with ADRD beyond infection and physical symptoms, and family caregivers have been crucial to maintaining care recipients’ health and quality of life. The experiences of Latino families with ADRD during the pandemic resembled those of the general population. These experiences included a lack of access to engaging activities and human contact, issues with the technology needed to replace in-person contacts, accelerated cognitive deterioration, and an impact on caregivers’ and care recipients’ mental health. However, this pandemic has revealed many of the barriers that Latino families with ADRD face, and in most cases, this pandemic has exacerbated previous barriers. These barriers included nutrition being affected by inefficiencies of the mailing system or financial insecurity; finances being affected by the pandemics’ impact on jobs typically held by Latino caregivers; and fatalism and personalism interacting with Latino individuals’ disproportionate risk to COVID-19 infection and death, which reduced health care use. These findings suggest the need for more equitable access, culturally appropriate and trustworthy content and delivery of health care and community services, as well as stronger financial and social supports for family caregivers.

## Supplementary material

10.2196/42211 Multimedia Appendix 1 Interview guides.

## Abbreviations

ADRD: Alzheimer disease and related dementias
PCP: primary care provider
