# Supplementary material for: Impact of the COVID-19 Pandemic on Latino Families With Alzheimer Disease and Related Dementias: Qualitative Interviews With Family Caregivers and Primary Care Providers
Source: JMIRx Med. 2024 Mar 8;5:e42211. doi: 10.2196/42211 (PMC11004515; doi:10.2196/42211)
Supplement: Multimedia Appendix 1 [file xmed-v5-e42211-s001.docx]

# Appendix 1. Interview guides

## Family caregivers

### Tell me about the two of you.

#### Prompts: Age, relationship, English/Spanish proficiency, PWD insurance, how long they know each other, profession, how long ago the PWD was diagnosed, where were they diagnosed, severity, main concern for either, currently related to the disease.

### Describe how you loved one was detected and diagnosed.

#### Prompts: Primary care vs specialists, Spanish speaking provider/interpreter, what triggered assessment, stage of disease, screening tools and their language/description, questions, labs, additional assessments, who administered assessments, who were assessments administered to (patient vs care partner), how was diagnosis communicated.

### Describe the dementia care and treatment services received by your loved one and yourself.

#### Prompts: Primary care vs specialists, Spanish speaking provider/interpreter, medications, behavioral interventions, what triggered assessment, followed up with assessments (how often, what tests were used, who were they administered to, feedback on follow-up), what treatment plan was offered, who participated in developing it, what domains did it address (dementia education, BPSD, safety, care transitions, internal or external referrals).

### What went well with these services and what could be improved about those services?

#### Prompts: language barriers, culturally appropriate, easy to follow, clear structure of plan, clear communication, feedback.

### How relevant to your culture as a Latino family were the services received?

### What are some of the additional challenges that should be considered in these services?

### How has the COVID-19 pandemic impacted your loved one and yourself?

### If the Alzheimer’s Disease Center provided your PCPs with dementia education, what aspects would you like them to be trained in?

#### Prompts: Covering how to best serve Latinos, duration, online vs in person, time of the day, topics, materials, protocols, group or individual, recorded or live, evaluation, venue, expertise of trainers, modality (lecture, caregiver/patient panels, skills practice), availability for continued training or consulting.

### If the Alzheimer’s Disease Center provided you with a dementia Navigator what would the optimal navigation services look like?

#### Prompts: Bilingual, at no cost to patient/clinic or CMS-covered, dementia education, caregiver well-being, home and driving safety, addressing BPSD, care transitions and community referrals, helping with appointments.

### What concerns would you have if we offered you these navigation services?

#### Prompts: Concerns about time constraints, practicality, burden.

## Primary care providers

### Tell me about yourself as a PCP.

#### Prompts: education as nurse, medical doctor, etc., ethnicity, Spanish language abilities, specialty, years in this profession, type of clinical institution, experience serving Latinos, experience serving people with dementia, distribution of Latino, older and dementia patients.

### Describe the typical service a patient 65 and older would receive in a yearly visit.

### What differences, if any, are there with Spanish or English-speaking Latinos?

#### Prompts: cutoffs, validated tools, interpreters, type of insurance, tailored information, assessments or treatments, different complaints.

### Tell me about someone who was recently diagnosed with dementia or MCI. What differences, if any, are there with Spanish or English-speaking Latinos?

#### Prompts: what stage (is it usually early or late stage), what triggered assessment, how was the assessment done, what tests were used, for what domains and who were they administered to, how was it communicated, how often will this person be reassessed, what treatment plan was offered, who participated in developing it, what domains did it address: dementia education, BPSD, safety, legal and financial assistance, care transitions, internal or external referrals.

### In general, how do you become aware that your patients have suspected dementia or cognitive impairment?

### What differences, if any, are there with Spanish or English-speaking Latinos?

#### Prompts: Patient discloses, care partner discloses, annual screening, accident.

### What steps do you take after you suspect cognitive impairment?

### What differences, if any, are there with Spanish or English-speaking Latinos?

#### Prompts: screening patient/care partner, referring to specialist, asking own questions to patient/care partner, diagnosis, labs, assessment of BPSD or functioning, cog testing, treatment, care, referral to resources, Navigator.

### Does your clinic or you follow a specific protocol for dementia detection, treatment and/or care? If so, would you mind sharing? Are there any particularities about the dementia services and clinic protocols you provide to your English and Spanish Latino patients?

### What are issues that come up around biological, psychological and social needs when you are working with Latino people with dementia/MCI and their families? To what extent do you feel your clinic is able to meet their needs?

#### Prompts: language barriers, family conflict, financial issues, lack of insurance, non-compliance, bad response to treatment, lack of resources in Spanish, lack of assessment materials in Spanish, lack of trust, fear of procedures, misconceptions.

### What are challenges to MCI and dementia detection and care experienced with Latino patients?

#### Prompts: language barriers, family conflict, financial issues, lack of insurance, non-compliance, bad response to treatment, lack of resources in Spanish, lack of assessment materials in Spanish, lack of trust, fear of procedures, misconceptions.

### How has the COVID-19 pandemic impacted the Latino families with dementia you serve?

### If the Alzheimer’s Disease Center provided PCPs with dementia education, what would the optimal education look like?

#### Prompts: Covering how to best serve Latinos, duration, online vs in person, time of the day, topics, materials, protocols, group or individual, recorded or live, evaluation, venue, expertise of trainers, modality (lecture, caregiver/patient panels, skills practice), availability for continued training or consulting.

### If the Alzheimer’s Disease Center provided PCPs with a dementia Navigator, what would the optimal navigation services look like?

#### Prompts: Bilingual, at no cost to patient/clinic or CMS-covered, dementia education, caregiver well-being, home and driving safety, addressing BPSD, care transitions and community referrals, helping with appointments.

### What concerns would you have if we offered you these dementia education and navigation services?

#### Prompts: Concerns about stealing patients, time constraints, practicality.
